# Use of propofol for prevention of post-delivery nausea during cesarean section: a double-blind, randomized, placebo-controlled trial

**DOI:** 10.1007/s00540-018-2549-x

**Published:** 2018-09-12

**Authors:** Kun Niu, Hui Liu, Ruo-Wen Chen, Qi-Wu Fang, Hui Wen, Su-Mei Guo, John P. Williams, Jian-Xiong An

**Affiliations:** 1 Department of Anesthesiology, Weifang Medical University, Weifang City, 261000 Shandong China; 2 Department of Anesthesiology, Pain Medicine and Critical Care Medicine, Aviation General Hospital of China Medical University and Beijing Institute of Translational Medicine, Chinese Academy of Sciences, Beiyuan Rd 3# Beijing, 100012 China; 3 Department of Pediatrics, Aviation General Hospital of China Medical University and Beijing Institute of Translational Medicine, Chinese Academy of Sciences, Beijing, 100012 China; 4 Department of Anesthesiology, University of Pittsburgh School of Medicine, Pittsburg, PA 15213 USA

**Keywords:** Cesarean section, Propofol, Post-delivery, Nausea and vomiting

## Abstract

**Purpose:**

Nausea and vomiting are common, undesirable symptoms during cesarean section. We conducted this study to assess the antiemetic properties of propofol for the prevention and immediate treatment of post-delivery nausea and vomiting during cesarean section under combined spinal–epidural anesthesia.

**Methods:**

Eighty women undergoing elective cesarean delivery under combined spinal–epidural anesthesia were randomized to receive either propofol at a plasma concentration of 1000 ng/mL or normal saline immediately after clamping of the umbilical cord. The incidence of post-delivery nausea and vomiting, patients requiring rescue antiemetic, bispectral index, sedation score, and the incidence of hypotension were assessed intraoperatively. Satisfaction and neonatal behavioral neurological assessments were evaluated postoperatively.

**Results:**

The incidence of nausea was significantly lower in the propofol group compared to the placebo group (25% versus 60%, *P* < 0.001). The incidence of retching and vomiting showed no significant difference between the two groups. Propofol 20 mg as a rescue antiemetic was significantly effective in both the groups. Satisfaction level of patients and obstetricians in the propofol group was higher than in the placebo group. There was no statistical difference in the incidence of hypotension between the two groups both pre- and post-delivery. There was no difference in postoperative neonatal behavioral neurological assessment between groups.

**Conclusion:**

Propofol at a plasma concentration of 1000 ng/mL significantly reduced the incidence of post-delivery nausea compared to placebo, but had no effect on reducing retching or vomiting episodes during cesarean section.

## Introduction

Intraoperative nausea and vomiting (IONV) are among the common undesirable symptoms in women undergoing spinal anesthesia for cesarean section [1, 2]. Besides causing patient discomfort, it interferes with surgery and increases procedure time, risk of bleeding, inadvertent surgical trauma, and risk of pulmonary aspiration of gastric contents [3].

In view of such high incidence of intraoperative post-delivery nausea and vomiting, several studies have made an effort to design both medication and non-medication therapies [1, 4–9], such as ondansetron, metoclopramide, droperidol, ginger, acupressure and acupuncture. Although these treatments have been proved to reduce the incidence of nausea and vomiting, many of these drugs were standard treatment for postoperative nausea and vomiting (PONV) rather reluctantly for parturient [2]. Ideally, one would prefer a more comfortable, safe and effective prophylaxis and immediate treatment of IONV occurrence for parturient.

Previous studies have confirmed that a bolus dose or a continuous infusion of propofol has direct antiemetic properties [2, 3, 10]. In addition, only a few clinical trials have demonstrated that infusion of propofol at a low dose (1.0 mg/kg/h) is effective in prevention of nausea and vomiting during and after cesarean section [11–13]. However, they did not provide the treatment details about parturient, who had experienced immediate nausea and vomiting, or evaluated the neonatal behavior associated with postoperative breastfeeding. We hypothesized that a continuous infusion of propofol, along with bolus doses for immediate control, might be effective and safe to decrease the incidence of IONV compared to placebo in parturients undergoing cesarean section.

## Methods

The study was conducted according to the Ethical Principles for Medical Research Involving Human Subjects outlined in the Declaration of Helsinki. This work was approved by the Ethical Committee of Aviation General Hospital (HK20161011) and registered at http://www.chictr.org.cn/index.aspx (ChiCTR-INR-16009539). All participants signed an informed consent form. Parturients with a singleton pregnancy, ASA physical status I or II, and receiving combined spinal–epidural anesthesia for elective cesarean section were included. We excluded patients with severe cardiac disease; insulin-dependent diabetes mellitus; severe impairment of hepatic or renal function; contraindications to regional anesthesia; history of allergic reactions to propofol or ondansetron; and those who had an antiemetic drug administered within 24 h prior to anesthesia. This single-center trial was conducted between October 2016 and February 2017 at Aviation General Hospital, Beijing, China. This manuscript adheres to the CONSORT guidelines.

Patients were randomly allocated by a random number generator system by research fellow A in a 1:1 allocation ratio to receive either normal saline (placebo group) or propofol (propofol group). Both patients and the anesthesiologists were blinded to randomization. Equal volumes of both 1% propofol (10 mg/mL, Xi’an Libang Pharmaceutical& Co., Ltd; Xi’an, Shaanxi Province, China) and 0.9% normal saline were drawn in a 20-mL syringe by research fellow A. The exterior color of each syringe was similar and indistinguishable by using a wrapped paper and the treatment was masked with opaque infusion lines. The parturient head site was covered by surgical drapes during the operation so they could not see the infusion line. After obtaining consent, patients were allocated to either the propofol or the placebo group by opening a sealed opaque envelope. Before the administration of anesthesia, research fellow B instructed each patient to report if they felt nausea at any time during the surgery.

All patients were administered 500–1000 mL of Lactated Ringer’s solution prior to the commencement of anesthesia. Standard monitoring included ECG, non-invasive blood pressure (BP), and pulse oximetry. Data on monitored parameters were collected by research fellow B. We used a combined spinal–epidural (CSE) anesthesia technique across the study groups: patients were placed in either the right or left lateral decubitus position, and skin infiltration with 2% lidocaine was carried out at the L2–L3 or L3–L4 interspace. A 16-gauge 80.00-mm AN-E epidural needle (Round-smooth; Tuoren Medical Device & Co., Ltd; Xinxiang, Henan Province, China) was inserted into the epidural space by an anesthesiologist through the loss of resistance technique using normal saline. Following this, a 25-gauge 113-mm AN-S Type II needle (Pencil Point; Tuoren Medical Device & Co., Ltd; Xinxiang, Henan Province, China) was advanced through the epidural needle until free flow of cerebrospinal fluid was obtained. Hyperbaric bupivacaine, 0.5% (a combination of 2.0 mL 0.75% bupivacaine and 1.0 mL 10% glucose solution), at a dose of 7.5–10 mg, was injected intrathecally. The epidural catheter was used during surgery to administer bolus of opiates if needed for inadequate analgesia and the catheter was removed at the end of the surgery. An adequate level of sensory block (T6 or above) was ensured by loss of sensation to pinprick before the commencement of surgery. After induction of anesthesia, patients were returned to the supine position with a 15-degree wedge under the right hip for displacement of the uterus to the left side, to avoid aortocaval compression. All patients received supplemental oxygen at the rate of 5 L/min via face mask. Non-invasive BP recordings were measured at 1-min intervals starting 1 min after the completion of intrathecal injection and continued till delivery of the newborn. Administration of Lactated Ringer’s solution, 10 mL/kg, commenced before anesthesia, was ceased approximately 5 min after the intrathecal injection (total infusion period, approximately 10 min).

Hypotension was defined as a decrease in systolic pressure by more than 20% from the baseline or an absolute value of less than 90 mmHg. Baseline blood pressure and heart rate were taken as the mean of the three recordings on the day prior to the surgery. Intravenous maintenance fluid, with Lactated Ringer’s solution, was infused at the rate of 2 mL/kg/h in both the groups. If hypotension persisted after a rapid fluid bolus, phenylephrine (100–150 µg bolus) was administered intravenously. Further hemodynamic management was at the discretion of the attending anesthesiologist.

After the baby was delivered, a continuous infusion of normal saline or propofol was commenced to reach a predicted plasma concentration of 1000 ng/mL through a computer-assisted continuous infusion device (Kelly Med™ Syringe Pump, KL-605T, Beijing, China) adjusted to patient age, sex, height and weight by research fellow B. Oxytocin (10 units intramuscularly, followed by an infusion of 20 units added to 500 mL Lactated Ringer’s solution) was routinely administrated. Apgar score at 1 min was evaluated by an experienced midwife.

Nausea was defined as a subjective sensation suggesting that a patient desires to vomit, retching as an expulsive effort not associated with the regurgitation of stomach contents, and vomiting as a forceful expulsion of gastric contents from the mouth [14]. Patients were instructed to report nausea based on an 11-point Verbal Rating Score (VRS), where 0 describes “no nausea” and 10 describes nausea “as bad as it could be”. Any score more than 0 was considered as nausea. Retching was considered as vomiting. Patients who developed significant nausea (VRS score ≥ 5), retching, or vomiting or requested a rescue agent were administered propofol 20 mg intravenously, followed by evaluation after 5 min using VRS. Symptoms were considered to have ceased if there was a reduction in VRS by at least 50%. If nausea and vomiting persisted, a further 20 mg of propofol was administered followed by reassessment 5 min later. Ondansetron 4 mg was administered intravenously if two doses of propofol, 20 mg each, failed to control nausea and vomiting. The incidence of IONV, VRS and patient requests for rescue antiemetic (20 mg propofol) were recorded immediately after clamping of the umbilical cord at 10, 20, 30, and at the end of the surgery by research fellow B, which was conducted according to Gan et al. [15].

The level of sedation was determined on a 5-point scale (0, alert; 1, aroused by voices; 2, aroused by gentle tactile stimulation; 3, aroused by vigorous stimulation; and 4, lack of responsiveness) [16]. Bispectral index (BIS) values were obtained and the median scores recorded every 10 min after the umbilical cord was clamped and cut. Data were recorded intraoperatively by downloading BIS values continuously to a laptop computer. Before anesthesia, the BIS sensor (BIS Quatro Sensor) was attached with four electrodes placed at an angle over the forehead of the patient, with values above 90 [17]. The incidence of nausea and vomiting after delivery, the need for a rescue antiemetic, BIS values, sedation scores, and the number of patients requiring intraoperative phenylephrine were assessed for the duration of surgery by research fellow B.

Patients were asked to rate their satisfaction level with the anesthetic experience and IONV control 24 h after the study using a 100-mm visual analog scale. Obstetricians were also asked to rate their satisfaction with surgical conditions associated with IONV using the same method by research fellow C. Any adverse events encountered were noted 24 h after surgery by the same research fellow who was blinded to the randomization. Neonatal behavioral neurological assessment (NBNA) was conducted by a pediatrician 24 h after commencement of breastfeeding, which contains 5 individual observations of behavior, passive tone, active tone, primary reflexes and general reactions. The total score of NBNA is 40; neonates with a total score of equal to or more than 37 are considered normal, while those with a score of less than 37 are considered to have a low NBNA [18].

### Statistical analysis

We based our power calculation on a pilot study, performed with a total of 60 cases, 28 in the placebo group and 32 in the propofol group. One of the primary outcome was the incidence of IONV. We found that without prophylaxis, more than 60% in the placebo group undergoing cesarean section experience at least one episode of nausea in the post-delivery period. The sample size was predetermined using power analysis based on the following assumptions: (1) the incidence of IONV would be 60% in the placebo group; and (2) a 40% reduction in the incidence of IONV (from 60 to 20%) by propofol would be considered clinically significant. The analysis showed that 28 patients per group would be sufficient to detect a difference with an α-error of 0.05 and a power (1-β) of 0.8. A larger number of patients, 40 patients per group, were enrolled to account for possible incomplete data collection or patient dropout (the potential patient dropout rate was 20%). Patient demographic data, NBNA, Apgar score, and satisfaction degree were analyzed by analysis of variance (ANOVA) with Bonferroni’s correction for multiple comparisons. Categorical variables, expressed as percentage with 95% confidence interval (95% CI), were compared using the Fisher exact test. The sedation score and BIS values were analyzed and compared by two-way ANOVA with repeated measures. Differences between groups were compared with an independent-sample *t* test. A *P* value < 0.05 was considered statistically significant. Values are given as means, standard deviations (SD), medians (ranges) or numbers (%). All statistical analysis was performed using SPSS software (version 19.0; SPSS Inc., IL, USA).

## Results

### Study patients

**Fig. 1:**
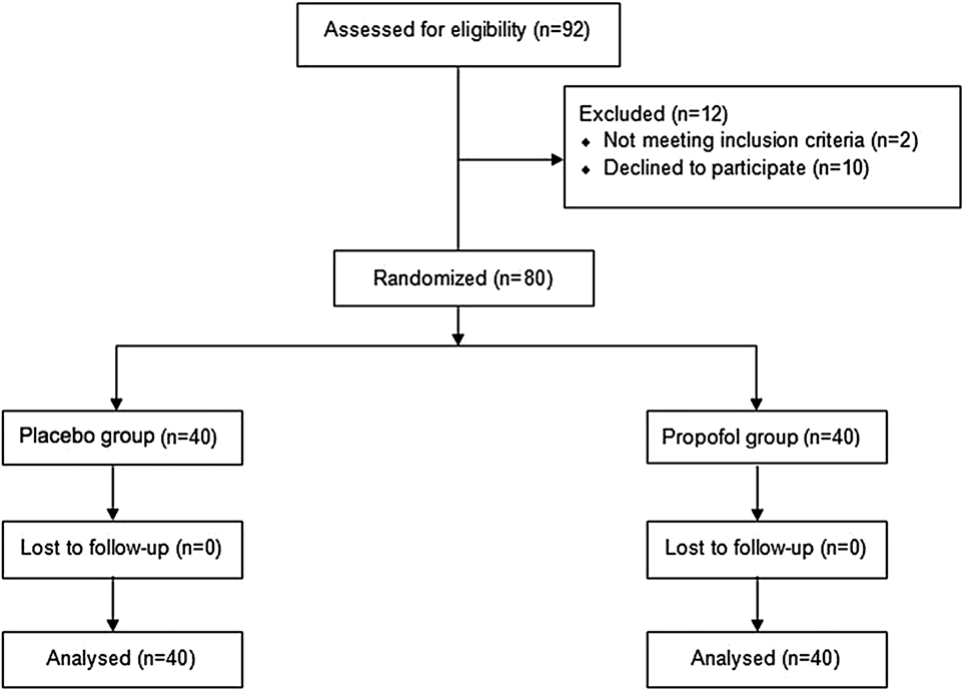
Participant flowchart according to CONSORT statement

Ninety-two patients were scheduled for cesarean section during the study period, out of which 12 patients were excluded (Fig. 1). The remaining 80 parturients completed the trial. Forty patients each were included in the propofol and the placebo group. Data were analyzed using an intent-to-treat analysis to ensure unbiased comparisons between treatment groups. There were no significant differences between groups in demographic, clinical and intraoperative characteristics (Tables 1, 2). No patients experienced motion sickness history. The total dose of the study medication ranged from 10.0 to 16.9 mL in the placebo group and 10.1–18.1 mL in the propofol group. No patients complained about pain during the surgery.

**Table 1:** Maternal and fetal characteristics

Variables	Placebo group (*n* = 40)	Propofol group (*n* = 40)	*P* value
Age (year)	31.7 ± 4.07	32.1 ± 4.63	0.060
Weight (kg)	72.2 ± 8.94	70.6 ± 6.01	0.304
Height (cm)	162 ± 4.56	161 ± 4.08	0.220
BMI (kg/m^2^)	27.3 ± 2.81	27.2 ± 2.37	0.832
Gestational age (week)	38.6 ± 0.868	38.5 ± 1.26	0.509
Baseline systolic pressure	123 ± 8.59	125 ± 6.12	0.225
Baseline diastolic pressure	79.9 ± 5.74	82.3 ± 6.11	0.068
Baseline heart rate	87.2 ± 12.5	91.8 ± 7.20	0.070
Fetal heart rate	139 ± 4.37	137 ± 6.09	0.151

Data are presented as mean (standard deviation)

Placebo group: patients receive continuous infusion of normal saline after clamping of the umbilical cord. Propofol group: patients receive continuous infusion of propofol after clamping of the umbilical cord

BMI body mass index

**Table 2:** Intraoperative characteristics

Variables	Placebo group (*n* = 40)	Propofol group (*n* = 40)	*P* value
Incidence of pre-delivery hypotension	35 (88)	32 (80)	0.833
Incidence of post-delivery hypotension	17 (43)	22 (55)	0.368
Exteriorization of the uterus	23 (58)	18 (45)	0.087
Abdominal irrigation	40 (100)	40 (100)	1.00
Skin incision to delivery (min)	6.18 ± 2.15	6.83 ± 2.82	0.239
Duration of surgery (min)	46.4 ± 7.87	43.9 ± 8.25	0.153
Dosage of study medication (mL)	13.6 ± 1.54	13.5 ± 2.34	0.787
Total phenylephrine used (mg)	0.880 ± 1.22	0.850 ± 0.921	0.895

Data are presented as mean (standard deviation) or *n* (%)

Dosage of study medication: the total dose of propofol or normal saline administered during the surgery

### Efficacy

**Fig. 2:**
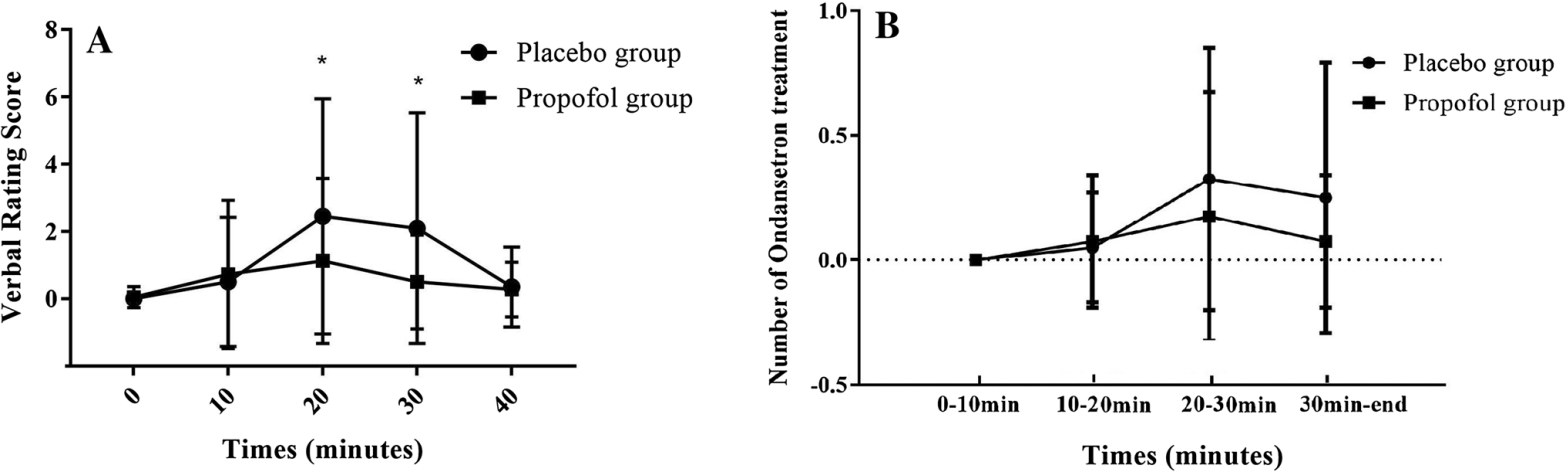
The Verbal Rating Score and the number of ondansetron treatment vs time in a linear scale. The values are presented as mean ± SD. **a** The Verbal Rating Score. There was a significant decrease in Verbal Rating Score (*P < 0.05) at 20 and 30 min after the baby was delivered in the propofol group compared with the placebo group. **b** The number of patients with ondansetron treatment. There was no difference in the administration of ondansetron treatment between two groups in every period after delivery

**Fig. 3:**
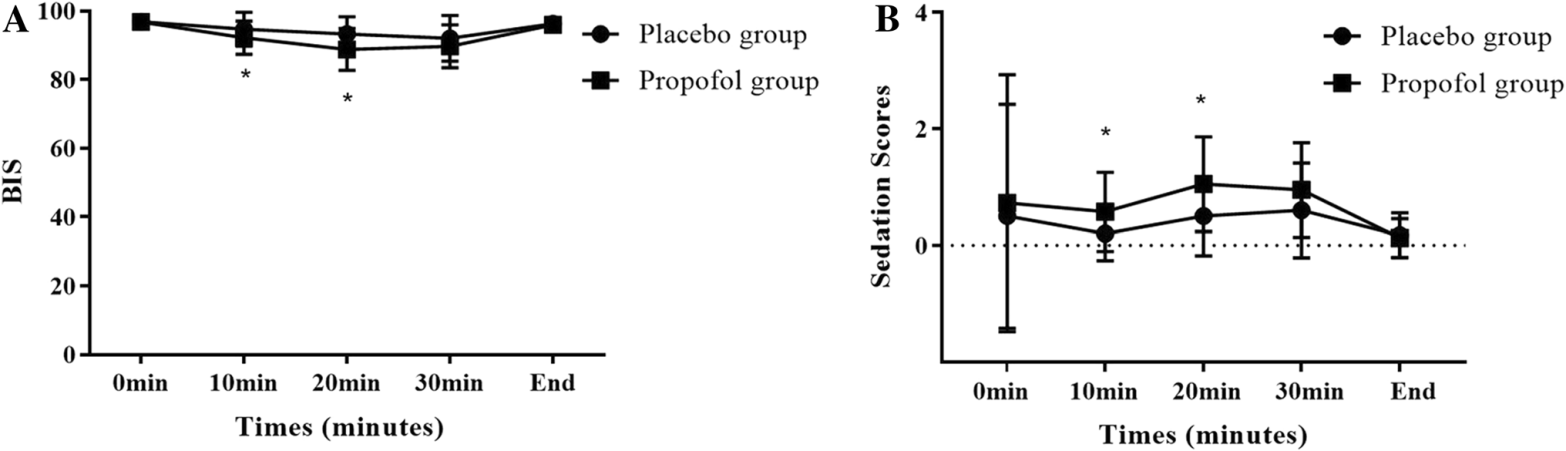
Bis value and sedation scores vs time in a linear scale. The values are presented as mean ± SD. **a** The median BIS values in the study groups and individual time intervals. There was a significant decrease in BIS value (*P < 0.05) at 10 and 20 min after the baby was delivered in the propofol group compared with the placebo group. *BIS* bispectral index. **b** The mean sedation scores. There was a significant decrease in sedation score (*P < 0.05) at 10 and 20 min after the baby was delivered in the propofol group compared with the placebo group

A significant difference was noted between the placebo group and the propofol group in terms of incidence of post-delivery nausea (relative risk 0.451; 95% CI 20.2–49.8%; *P* < 0.001); however, no difference was noted between these two groups in terms of incidence of retching and vomiting (relative risk 0.459; 95% CI, 3.93–26.1%; *P* = 0.112), as shown in Table 3. The difference in VRS between the propofol group and the placebo group was apparent: 20 min and 30 min after delivery (20 min: *P* = 0.041; 30 min: *P* = 0.01) (Fig. 2a). The number of patients requiring ondansetron was 4 in the propofol group and 9 in the placebo group (relative risk 0.573; 95% CI, 2.25–22.75%; *P* = 0.411) (Table 3). No difference was found in the administration of ondansetron treatment between the two groups in every period after delivery (*P* > 0.05) (Fig. 2b). Both patients and obstetricians in the propofol group expressed a higher degree of satisfaction (patients: 95% CI − 1.08 to − 0.212; *P* = 0.005; obstetricians: 95% CI − 2.84 to − 1.26; *P* < 0.001). BIS values were significantly lower in the propofol group compared to the placebo group 10 min and 20 min after delivery: 92.2 ± 4.96 vs 94.7 ± 4.90 (95% CI 0.511–4.54; *P* = 0.015) and 88.8 ± 6.11 vs 93.3 ± 5.05 (95% CI 1.99–6.95; *P* = 0.001), respectively (Fig. 3a). Sedation scores of patients were significantly higher in the propofol group compared to the placebo group 10 min and 20 min after delivery: 0.575 ± 0.674 vs 0.200 ± 0.464 (95% CI − 0.623 to − 0.127; *P* = 0.004) and 1.05 ± 0.814 vs 0.500 ± 0.679 (95% CI − 0.881 to − 0.219; *P* = 0.002) (Fig. 3b).

**Table 3:** Incidence of nausea and vomiting in the placebo and propofol groups

	Placebo group (*n* = 40)	Propofol group (*n* = 40)	*P* value
Intraoperative nausea^a^	24 (60)	10 (25)^c^	< 0.001
Intraoperative retching and vomiting^b^	9 (23)	3 (8)	0.112
IONV initial treatment	18 (45)	9 (23)^c^	0.033
Relief after initial treatment	16 (89)	7 (78)	0.444
IONV additional treatment	2 (11)	2 (22)	0.444
IONV ondansetron treatment	9 (56)	4 (57)	0.411

Data are presented as number (%)

^a^Nausea: patients with nausea score more than 0 during continuous infusion of propofol or normal saline

^b^Retching and vomiting: patients experiencing any retching or vomiting during continuous infusion of propofol or normal saline

^c^Statistically significant

### Safety

Patients were followed up for adverse effects including hypotension, excessive sedation, uterine atony and respiratory depression; no adverse effects were reported. Fetal outcomes including Apgar score and NBNA after 24 h of commencement of breastfeeding were not different between the placebo and the propofol groups (Table 4).

**Table 4:** Fetal outcomes

Variables	Placebo group (*n* = 40)	Propofol group (*n* = 40)	*P* value
Apgar score at 1 min	10 (9–10)	10 (10–10)	0.308
Apgar score at 5 min	10 (10–10)	10 (10–10)	1.00
Breast feeding	40 (100)	40 (100)	1.00
NBNA score			
Total score	40 (39–40)	40 (38–40)	0.344
Behavior	8 (8–8)	8 (8–8)	0.261
Passive tone	6 (6–6)	6 (6–6)	0.610
Active tone	6 (6–6)	6 (6–6)	0.951
Primary reflexes	12 (11–12)	12 (11–12)	0.230
General reactions	8 (8–8)	8 (7–8)	0.815

Data are presented as medium (range)

## Discussion

Despite that continuous infusion of propofol has known efficacy in preventing both nausea and vomiting for parturients during the cesarean section [11–13], we found that the number of patients who experienced nausea was significantly lower in the propofol group compared to the placebo group; however, the incidence of vomiting was not different between groups.

A recent study reported that the incidence of postoperative nausea and vomiting (PONV) in cesarean section was 24% and 14%, respectively [19]. Compared to the plethora of studies conducted on the PONV, little attention has been paid to the intraoperative nausea and vomiting (IONV). The incidence of IONV is variable and in some extreme cases up to 80% [1]. The risk factors of PONV include being female, nonsmoker, opioid use, history of PONV or motion sickness. Parturients often meet at least two of these factors with their gender and the nonsmoker background. Besides, pregnancy itself is associated with nausea and vomiting linked with the reduced tone of the esophagogastric junction and increased intraabdominal pressure due to hormonal changes during pregnancy [2]. Other factors including a mass of blood loss in a few minutes reducing perfusion of the chemoreceptor trigger zone where the vomiting center is located in the medulla oblongata may result in IONV. Surgical stimuli as well as uterus exteriorization may cause IONV by activating afferent vagal afferences [20]. Besides, uterotonic agents, traditionally administrated after delivery of the baby, may also lead to the high incidence of IONV due to the hypotensive effect [2].

Traditional prophylactic agents such as metoclopramide and droperidol, which were limited to parturients undergoing cesarean section out of a fear of neonatal outcome. Moreover, such treatments may not completely avoid emetic symptoms during operation when an urgent response is warranted. Metoclopramide, a dopamine antagonist, acts on the D-2 receptors in the chemoreceptor trigger zone. However, side effects including sedation, agitation, extra-pyramidal effects and akathisia have been reported in previous studies [4, 21]. Droperidol as a prophylaxis medication is most effective in the prevention of PONV [5]. However, due to the 2001 black box warning [22], it has been limited in many countries. Recently, Amouee and colleagues demonstrated that ginger decreased the severity of nausea and vomiting during and after cesarean section [6]. Kalava and colleagues demonstrated that preoperative ginger capsule containing 1 g of ginger powder reduced the severity, but not the incidence of IONV [7]. Several studies on acupressure and acupuncture have shown benefits in the prevention of nausea and vomiting during cesarean section; however, these techniques were of doubtful efficacy and resulted in adverse effects such as localized discomfort, itching or hand swelling [8, 9].

Gan et al. found that plasma concentrations of 343 ng/mL and 592 ng/mL of propofol reduced nausea in 50% and 90% of patients, respectively, using a computer-assisted continuous infusion device in the PACU [23]. Subsequently, they have demonstrated that propofol at a demand dose of 20 mg was effective in managing PONV with shorter PACU stay and higher degree of patient satisfaction [15]. Recent studies used propofol as a 20 mg bolus and 1.0 mg/kg/h as a continuous infusion immediately after clamping of the umbilical cord during the cesarean section [11–13]. Although these studies showed that propofol could reduce the incidence of nausea and vomiting, they either failed to prove the safety of propofol in breastfed neonates or provide an urgent treatment for the sudden-onset nausea. Moreover, the use of target-controlled infusion and BIS monitor has not been reported in these studies [11–13].

The emetic stimuli could arise from several pathways—vestibular, cerebral cortex, area postrema and gastrointestinal tract via the vagus nerve to the emetic center, which is believed to reside in the ventrolateral reticulate structure [24]. According to an animal study, the antiemetic action of propofol is attributed to a decrease in 5-HT levels in the area postrema and probably through its action on the GABA receptors [25]. Thus, propofol may reduce the incidence of nausea by inhibiting and blocking signal transduction in the area postrema; it may have no effect on vomiting because of signaling from other pathways. The short duration of action of propofol may lead to a relative lack of efficacy.

A bolus dose of propofol 20 mg is effective as a rescue antiemetic. Sixteen of 18 in the placebo group and 7 of 9 in the propofol group obtained reliable relief after receiving propofol 20 mg. However, there was concern that high blood levels of propofol might lead to excessive sedation. The ideal dose should provide an optimal plasma concentration of propofol that prevents nausea and vomiting, but does not result in significant respiratory depression or excessive sedation. Patients receiving propofol were slightly more sedated, as observed from their lower BIS values compared to those who received normal saline; however, there was no noticeable respiratory depression. The satisfaction level of patients and obstetricians in the propofol group was higher than in the placebo group, suggesting that patients receiving light sedation during surgery were more comfortable than those who were awake and possibly anxious; in addition, escorting these patients becomes more convenient. The rates of change in blood pressure and oxygen saturation levels were not significantly different between groups. We found no significant difference between groups with regard to NBNA at 24 h following drug administration, suggesting that the propofol dose used was safe for lactating women and babies, which is consistent with other reports [26].

Our study has several limitations. First, we did not assess the incidence of nausea and vomiting before delivery; however, we prevented hypotension, which is known to cause nausea and vomiting. Second, we used the same infusion dose of propofol in all our patients; no attempt was made to compare different infusion doses. Third, we did not use intrathecal opioid in this study because of the fear of the addictive properties of opioid from obstetricians and public. Koju et al. [27] have reported that intrathecal administration of opioids led to pruritus, nausea and vomiting after cesarean delivery, which would confuse the study outcome. Furthermore, we noticed that several patients complained about injection pain caused by propofol, however we did not use lidocaine because it was reported that the propofol injection caused pain in approximately 25% of patients, even if 30 mg lidocaine was administered [28].

In this study, we found that a continuous infusion of propofol, targeted to a plasma concentration of 1000 ng/mL, which required less often a propofol 20 mg bolus, prevents post-delivery nausea and this group reacted better to this bolus, although it did not prevent vomiting. Therefore, a continuous infusion of propofol combined with a 20 mg bolus or with adjuvant antiemetic drugs may be a suitable approach for decreasing the incidence of intraoperative nausea, but is ineffective for vomiting during cesarean section.
